# Forecasting large aftershocks within one day after the main shock

**DOI:** 10.1038/srep02218

**Published:** 2013-07-17

**Authors:** Takahiro Omi, Yosihiko Ogata, Yoshito Hirata, Kazuyuki Aihara

**Affiliations:** 1 FIRST, Aihara Innovative Mathematical Modelling Project, Japan Science and Technology Agency, Kawaguchi, Saitama 332-0012, Japan; 2 Institute of Industrial Science, The University of Tokyo, 4-6-1 Komaba, Meguro-ku, Tokyo 153-8505, Japan; 3 The Institute of Statistical Mathematics, Tachikawa, Tokyo 190-8562, Japan

## Abstract

Forecasting the aftershock probability has been performed by the authorities to mitigate hazards in the disaster area after a main shock. However, despite the fact that most of large aftershocks occur within a day from the main shock, the operational forecasting has been very difficult during this time-period due to incomplete recording of early aftershocks. Here we propose a real-time method for efficiently forecasting the occurrence rates of potential aftershocks using systematically incomplete observations that are available in a few hours after the main shocks. We demonstrate the method's utility by retrospective early forecasting of the aftershock activity of the 2011 Tohoku-Oki Earthquake of M9.0 in Japan. Furthermore, we compare the results by the real-time data with the compiled preliminary data to examine robustness of the present method for the aftershocks of a recent inland earthquake in Japan.

A vast number of aftershocks occur following a large earthquake. Especially, in the first 24 h after the main shock, a probability of a large aftershock that possibly causes the secondary disaster in and around the focal area is high. In this period, more than a half of the strong aftershocks that occur in the first months after the main shock, in fact, occur, and further most of the largest aftershocks occur in this period^1^,^2^. Hence, it is desired to give a probability forecast of a large aftershock as soon as possible after the main shock. On the other hand, despite the effort to improve early forecast^3^,^4^, immediate forecast has been considerably difficult^5^,^6^. Actually, operational probability forecasts by the Japan Meteorological Agency (JMA) start after more than 24 hours of the main shock^6^. This is mainly due to the substantial deficiency of data. Many aftershocks occurring shortly after the main shock are missing from the seismic records due to overlapping of seismograms^6^,^7^,^8^,^9^,^10^. Further difficulty is the property that the number of strong aftershocks considerably differs depending on a main shock even if the magnitude of the main shock is similar^2^.

Here we propose a method for forecasting underlying aftershock activity that includes missing aftershocks, using the incomplete observations that are available a few hours after the main shock. In this method, we introduce a statistical model of the incompletely detected aftershocks. This model is then combined with the model adopted by Reasenberg and Jones^3^. This procedure enables us to give the forecast without relying on the generic model based on the main shock size. The usefulness of the proposed method in forecasting aftershock activity within 24 h after the main shock is demonstrated by retrospective early forecasting of the aftershock activity of the 2011 Tohoku-Oki Earthquake of M9.0 in Japan by analysing the data from the National Earthquake Information Center/Preliminary Determination of Epicenters (NEIC/PDE) catalogue (Fig. 1). We also analyse the aftershocks of the M6.3 earthquake on Feb. 25, 2013 in Nikko, central Honshu, Japan, by using the real-time data of the High Sensitivity Seismograph Network (Hi-net) operated by National Research Institute for Earth Science and Disaster Prevention (NIED)^11^, and show the effectiveness of the present method in a realistic situation.

## Results

### Estimating the forecasting model from observed data of incompletely detected earthquakes

For the occurrence rate of underlying aftershocks of magnitude *M* at elapsed time *t* from the main shock, we employ the following model^3^: 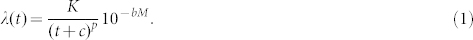
Here the parameters *K*, *c*, and *p* of the Omori–Utsu (O-U) formula for aftershock decay^7^,^12^,^13^ and the *b*-value of the Gutenberg–Richter (G-R) formula for the magnitude frequency relation^14^ are constants to be estimated. Here, the parameter *K*, which controls the number of aftershocks, greatly depends on individual aftershock sequence, even if the magnitudes of the main shocks are almost the same: For example, the 2004 Chuetsu earthquake and the 2007 Chuetsu-oki earthquake in Japan, which are about 40 km apart, have the same magnitudes of M6.8, but the numbers of their aftershocks of *M* ≥ 4.0 differ by 6–7 times^2^. Hence, this parameter is particularly crucial for the early forecast, but has been difficult to estimate in an early period^5^,^6^.

Given the estimate of the parameters of the forecasting model (1), the occurrence rate *λ*(*t*) of aftershocks with magnitude *M* ≥ *M_p_* is calculated as 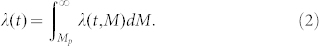
The method to calculate the 95% predictive interval of the empirical occurrence rate is described in Sec. S1 in Supplementary information.

**Figure 1:**
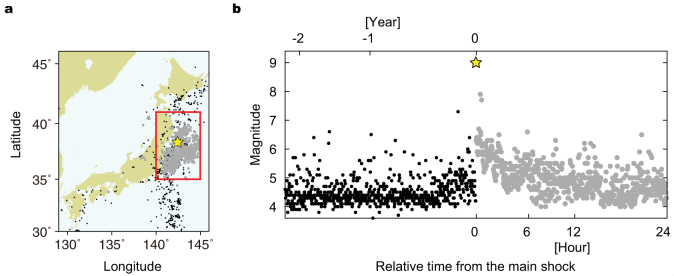
Observed earthquakes in Japan before and after the 2011 Tohoku-Oki earthquake of M9.0. (a) Epicenters and (b) time versus magnitude of earthquakes that occurred in Japan according to PDE/NEIC catalogue. Grey (black) closed circles represent earthquakes within one day after (within two years before) the main shock. The star indicates the main shock. The inner rectangular in (a) represents the aftershock area, and the data in this region is used for the forecasting experiment. Clearly many small aftershocks are absent just after the main shock [see (b)]. The map in Fig. 1a was generated by using GMT (Generic Mapping Tool).

One usually estimates these model parameters from observed data with magnitude greater than selected threshold^15^,^16^,^17^, avoiding the early period of incompletely detected aftershocks (Fig. 1). In this study, the parameters have to be estimated from such incompletely detected aftershocks in the early period for making an early forecasting. For the purpose, we must consider the statistical feature of incompletely detected aftershocks. To model the incomplete detection of earthquakes, previous studies^18^,^19^,^20^,^21^ introduced the detection rate function of magnitude, the probability at which each underlying earthquake is detected. The detection rate of an earthquake clearly depends on its magnitude such that smaller (larger) earthquakes are detected with a lower (higher) probability. The detection rate function of the magnitude is suitably represented by the cumulative distribution function of the normal distribution Φ(*M*|*μ*,*σ*), 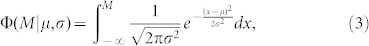
where *μ* represents the magnitude with a 50% detection rate, and *σ* represents a partially detected magnitude range^18^,^19^,^20^,^21^. By means of suitable estimate of the detection rate function, we can estimate the occurrence rates of the underlying earthquakes from given data of incompletely detected earthquakes.

**Figure 2:**
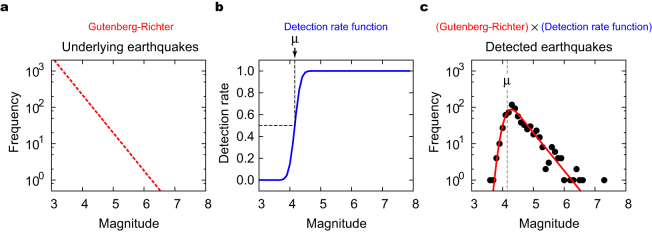
Statistical model of observed magnitude frequency distribution. (a) Assumed G-R formula for magnitude distribution of underlying earthquakes. (b) Detection rate function modelled as cumulative distribution function of normal distribution. The parameter *μ* represents the magnitude with a 50% detection rate. (c) Magnitude distribution (red solid line) of detected earthquakes, given as the product of the G-R formula and the detection rate function. Closed circles represent the empirical magnitude frequency distribution for earthquakes that occurred before the main shock in Japan (black closed circles in Fig. 1b).

The detection rate function and the *b*-value of the G-R formula can be estimated simultaneously. For homogeneously detected earthquakes, the observed magnitude distribution *p*(*M*|*β*,*μ*,*σ*) is modelled as a product of the underlying magnitude distribution *m*(*M*) = 10^−*bM*^ (the G-R formula) and the detection rate function Φ(*M*|*μ*,*σ*) (Fig. 2), given as 
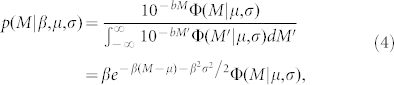
where *β* = *b*log_e_10. The parameters are estimated by maximizing the log likelihood function 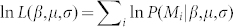, where ‘ln’ represents the natural logarithm, and {*M_i_*} is a series of observed magnitudes. Figure 2 demonstrates a good fit of the model to the observed data for homogeneously detected earthquakes in the Japan region before the 2011 Tohoku-Oki earthquake of M9.0.

However, the detection rate of aftershocks substantially varies in time: it is quite low immediately after the main shock and recovers to the normal level with time. To account for this, we assume that the parameter *μ*, which represents the magnitude with a 50% detection rate (Fig. 2b), is a function of the elapsed time. In other words, the magnitude distribution moves in time, and the parameter *μ*(*t*) specifies the position of the magnitude distribution at each elapsed time *t* after the main shock. Here, we assume that the *b*-value is constant throughout the entire aftershock sequence^7^,^22^,^23^. To make optimally adaptive estimation of the time-dependent parameter *μ*(*t*), we develop a state-space model for an objective Bayesian inference^24^,^25^,^26^. In addition, we employ a Gaussian prior probability distribution of the *b*-value for robust estimation using short data from an early period. The method is described in detail in Methods.

Next, given the estimate of the *b*-value and detection rate function, we can estimate the parameters *K*, *c*, and *p* of the Omori–Utsu formula for the underlying aftershocks^27^. The occurrence rate *ν*(*t,M*) of detected aftershocks is given by the product of the rate *λ*(*t,M*) of the underlying aftershocks in equation (1), and the estimated detection rate function *Φ*(*M|μ,σ*) in (3) as follows: 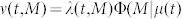. This leads to the log likelihood function of the parameters *K*, *c*, and *p*, given as 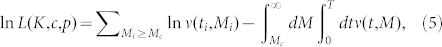
where *t_i_* and *M_i_* are the occurrence time and magnitude of the *i-*th aftershock observed in a learning period of duration *T* after the main shock, respectively, and we fit the model to detected aftershocks with magnitude *M* ≥ *M_c_*.

The details of the methods and procedures in estimation and forecasting are described below and supplementary documents. The flow of these procedures is summarized in Table 1.

### Forecast experiment of the aftershocks of the 2011 Tohoku-oki earthquake M9.0 in Japan

**Figure 3:**
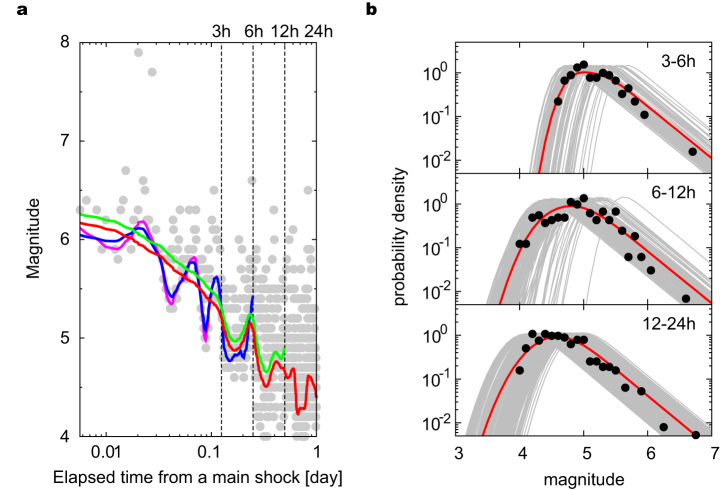
Estimation of time-dependent detection rate. (a) Estimates of *μ*(*t*) for learning periods of 3 h, 6 h, 12 h, and 24 h after the main shock are represented by magenta, blue, green, and red curves, respectively. (b) Closed circles represent normalized magnitude histograms of observed aftershocks. Each grey curve represents the instantaneous magnitude distribution predicted at the time when each earthquake occurs, and red curves represent their average for each time window.

Figure 3a shows the optimal estimation of the time-dependent parameter *μ*(*t*) for various periods until the time to forecast. The estimated *b*-values and other parameters are listed in Table 2. In a previous study^27^, *μ*(*t*) was assumed to be a parametric model of a monotonically decreasing function of time. However, the estimated *μ*(*t*) includes some oscillations that are scarcely realized by such a simple parametric model. A careful look at Fig. 3a suggests that *μ*(*t*) rose steeply after some large aftershocks: indeed, large aftershocks lower the detection rate again. The oscillation is, therefore, not an artefact of the estimation method. Hence, we take a state-space approach to adapt to such changes in the detection rate. To demonstrate that the present estimate is useful, we compare the empirical magnitude frequency of the observed aftershocks with that predicted by the model in various time windows (Fig. 3b). Figure 3b shows snap shots of real-time estimation of time-varying magnitude distribution, based on the adaptive estimation of the parameter *μ*(*t*) at each time when an aftershock has been detected. Figure 3b demonstrates the good fit of the predictive distribution to the data, validating the proposed procedure.

**Figure 4:**
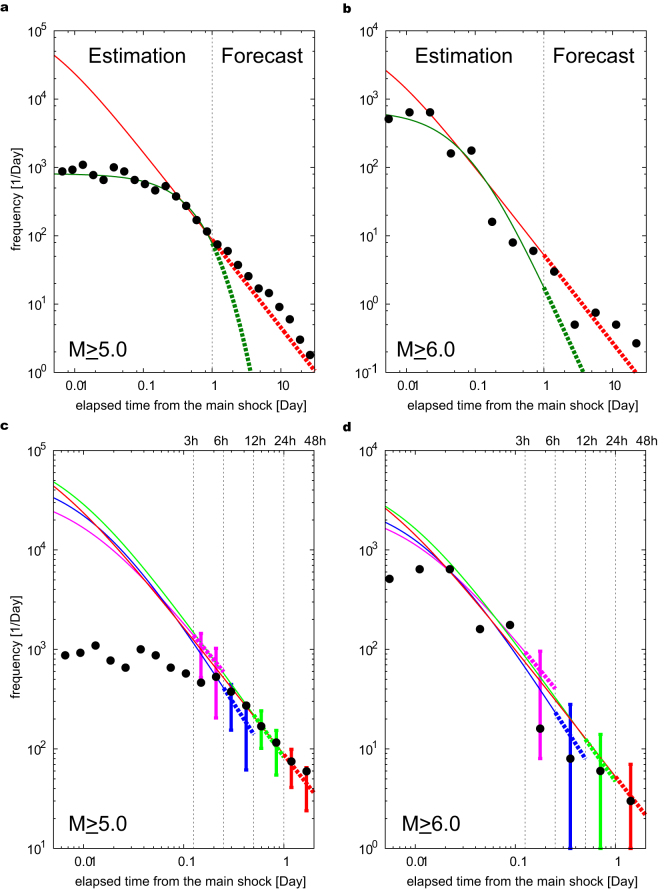
Forecast of underlying aftershocks and observed aftershocks. (a and b) Occurrence rates of underlying aftershocks with magnitude (a) *M* ≥ 5.0 and (b) *M* ≥ 6.0, estimated for the learning period of one day after main shock (red solid curve) and forecasted for the following 30 days (red dashed curve). Solid (dashed) dark green curves are estimates (forecasts) obtained by directly fitting the Omori–Utsu formula to the detected aftershocks. Closed circles represent empirical occurrence rates of observed aftershocks. (c and d) Occurrence rates of underlying aftershocks with magnitude (c) *M* ≥ 5.0 and (d) *M* ≥ 6.0, estimated for a learning period (solid curve) and forecasted for the following period of the same duration (dashed curve). Learning periods are 3 (magenta), 6 (blue), 12 (green), and 24 (red) h after the main shock. Bars indicate 95% predictive intervals of empirical occurrence rates (see Supplementary Information).

Given the estimate of the detection rate function and the G-R formula, we next obtain the maximum likelihood estimates *K*, *c*, and *p* of the O-U formula for the underlying aftershock activity. These estimates are listed in Table 2. Then, we forecast the occurrence rates of future underlying aftershocks with magnitude *M* ≥ 5.0 and 6.0 by using the model in equation (1). The forecasts based on observations made within only one day after the main shock agree well with the empirical occurrence rates of the observed aftershocks in the following 30 days (red lines in Figs. 4a and b). On the other hand, one may suspect that direct application of the O-U formula to the observed data could work well with a large *c*-value, which could compensate for the missing aftershocks in the early stage^7^. In order to examine this, we obtained these fits and their forecasts (green lines in Figs. 4a and b). In contrast to the proposed method, in which the detection rate is taken into account, these forecasts clearly deviate from the later observations in spite of its good fit to the observed data in the learning period (within one day after the main shock). The result demonstrates the importance of taking into account the detection rate in the analysis. Furthermore, this method forecasts well even for a learning period shorter than 24 h. Figures 4c and d show that the occurrence rates of the underlying aftershocks within 24 h of the main shock were forecasted very well on the basis of the observations in the first 3 h period and longer, despite the highly incomplete aftershock detection. Figures 4c and d also show the robustness of our estimates of the underlying occurrence rates during the learning periods, although the estimated parameters seem to be different depending on the learning period within 24 hours, which is due to the correlations between the parameters (such as *p*- and *c*-values) under relatively small sample sizes. Hence, we conclude that the present method is effective for forecasting the aftershock activity within 24 h after the main shock. Note that the rate of underlying aftershocks decays for even less than 0.01 day (15 minutes) in contrast to the rate of detected aftershocks (Figs. 4c and d). This is consistent with the recent study^28^ that carefully examined waveforms to identify aftershocks.

### Forecast experiment of the aftershocks of the strong inland earthquake in Japan using real-time data

Next we compare our results by the data from JMA preliminary catalog (open of 1–2 days delay) with the real-time data of the High Sensitivity Seismograph Network (Hi-net) operated by National Research Institute for Earth Science and Disaster Prevention (NIED)^11^. The Hi-net provides the data of earthquakes that were automatically detected, and also their original records are partly used for compiling JMA hypocenter catalog. We consider the aftershocks of the M6.3 earthquake on Feb. 25, 2013 in Nikko, central Honshu, Japan.

Supplementary Figures S1 and S3 show the estimation of the time-varying detection rate for the Hi-net catalog and JMA catalog, respectively. Supplementary Figs. S2 and S4 show the predicted frequency with the superimposed empirical frequency for the Hi-net catalog and JMA catalog, respectively. Although the Hi-net catalog contains less events than the JMA catalog (Supplementary Figs. S1a and S3a), the changing aftershock magnitude distribution within the 24 h can be suitably predicted based on the Hi-net catalog as well as the analysis based on the JMA data (see Supplementary Figs. S1b and S3b). Furthermore, it can appropriately forecast the aftershock activity within 24 h after the main shock by our procedure, indicating the usefulness of the proposed method for the early forecasting in a realistic situation (see Supplementary Figs. S2c and d). However, the 1-month ahead forecast using the Hi-net data slightly deviates from the future observation (Supplementary Figs. S2a and b) in contrast to the forecast using the JMA catalog (Supplementary Figs. S4a and b). Note that, because we only have the data during two days after the main shock for the Hi-net catalog, we have used the JMA catalog for the following period to compile Supplementary Figs. 2a and b, but the forecast model is constructed solely based on the data from the Hi-net. The estimated parameters for the Hi-net catalog and the JMA catalog are respectively listed in Supplementary Tables S1 and S2.

## Discussion

In this paper, we have proposed a method for forecasting underlying aftershock activity from the observed data of incompletely detected earthquakes. In our method, the time-varying detection rate is adaptively estimated by using the state-space model, and then this model is combined with the forecasting model employed by Reasenberg and Jones^3^. We have shown that our method can be successfully applied to the retrospective early forecasting of aftershocks of the 2011 Tohoku-oki earthquake. Furthermore, we also show that our method is effective and robust in early forecasting even for the real-time data, by analysing the aftershocks of the recent strong inland earthquake in Japan.

One might wonder whether we can employ a generic model for an early forecast, which has the parameters corresponding to those of the standard aftershock activity in a certain area. Indeed, generic *c* and *p*-values are not so sensitive to work robustly for a short period of a few days. However, the parameter *K* represents the individual feature of aftershocks in the focal area besides the main shock magnitude; in fact, it has been shown that cumulative numbers of aftershocks for the first 30 days differs in the order of 10 times even for the main shocks of similar magnitude of M7 class^2^. Due to the deficient aftershocks in the early period, we have to wait a couple of days to assess a suitable *K*-value for a generic model. This is a reason why early operational forecast has not been achieved by the authorities. In contrast, the present study aims to directly estimate the model parameters from the systematically incomplete data of the early period by considering the detection rate of earthquakes.

The O-U formula well represents aftershock activity unless there are any conspicuous secondary and successive aftershocks that followed a significantly large aftershock. However, aftershock sequences in general are not always accurately represented by the O-U formula^15^. Hence, for a longer period, it would be useful to extend the present forecasting method to include statistical models such as the ETAS model^29^. The extension is our important future problem.

Although the most of strong aftershocks occur within one day after the main shock, some largest aftershocks occur days or months after the main shock. Our model based on the O-U formula gives a small probability according to the G-R law for such events. A large aftershock sometimes accompanies precursory anomalous activity of aftershocks^30^. Thus the probability gain of such forecast might be improved by considering the anomaly of aftershock activity.

In our procedure, we have used cumulative function of a Normal distribution for the detection rate function of magnitude. This function works well if the hypocenter detection is based on an enough number of homogeneously located seismic stations^18^,^19^,^20^. There may be other possible detection functions, such as the logistic function^19^ and the exponential detection function^21^, dependent on configuration of seismic networks.

## Methods

### Data

We have first used the catalogue of the Weekly Listing version of Preliminary Determination of Epicenters (PDE-W) from the NEIC to analyse the aftershock sequence of the 2011 Tohoku-Oki Earthquake of M9.0 in Japan (Fig. 1). The data were obtained from the FTP website of the USGS (http://earthquake.usgs.gov/earthquakes/eqarchives/epic/), but one can use the real-time version, ‘Current Worldwide Earthquake List' (http://earthquake.usgs.gov/earthquakes/map/). The catalogue of the Japan Meteorological Agency (JMA) is typically used to study the seismicity around Japan because of its overall high detection of smaller earthquakes. However, in this particular case, the detection rates of aftershocks from a very wide area (Fig. 1) are spatially heterogeneous^19^, mainly depending on the distance from the east coast of northern Honshu because the JMA network is located in the inland area. On the other hand, the global NEIC network enables spatially homogeneous detection of aftershocks, although the stations are much sparser than those of the JMA. Since our goal is to provide a forecasting method that will work in real time, the forecasting method should be tested using either real-time observations or the earliest quasi-real-time observations. Therefore, we tested the proposed methods using the NEIC-PDE catalogue.

To analyse the aftershock sequence the M6.3 earthquake on Feb. 25, 2013 in Nikko, central Honshu, Japan, we have used the High Sensitivity Seismograph Network (Hi-net) catalog and the Japan Meteorological Agency (JMA) catalog. For the analysis of the aftershocks of the Nikko earthquake, the data is considered to be spatially homogeneous because the aftershock region is limited to the narrow area.

### Magnitude frequency model with time-dependent detection rates

In the setting described above, the magnitude of each earthquake can be considered a random realization of the hidden variable *μ*(*t*), and the noise is controlled by the hyper-parameters *β* and *σ*. A state-space model provides a useful framework for estimating a smooth profile of the hidden variable *μ*(*t*) from an observed magnitude sequence {*M_i_*}^24^,^25^,^26^.

For simplicity, we assume that *μ*(*t*) is a step function that changes upward or downward when each earthquake occurs; that is, *μ*(*t*) = *μ_i_* for the time interval *t_i_* ≤ *t* < *t_i_*_+1_ (*i* = 1, 2, …, *N*), where *t_i_* is the occurrence time of the *i-*th aftershock detected in an estimation period [0, *T*], and *N* is the number of detected aftershocks. Thus, we will estimate a sequence of the parameters 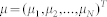. Given a sequence of magnitudes 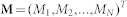 of detected earthquakes, the likelihood function is rewritten as 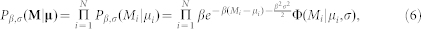
where 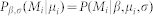 in equation (4). Because the model contains parameter 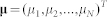, the maximum likelihood method gives a rough estimate of *μ*(*t*). Hence, we assume a prior distribution 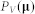 of a smoothness constraint for *μ*(*t*) that penalizes the second difference of **μ** and is given by 
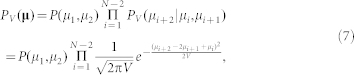
where *V* is a hyper-parameter that controls the degree of smoothness of *μ*(*t*). We assume that *μ*_1_ and *μ*_2_ obey the uniform prior; that is, 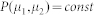.

From Bayes' rule, the posterior function 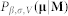 of **μ** given the observed magnitudes **M** is given by 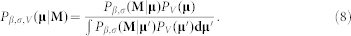
Our goal is to obtain the posterior mode 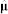 such that 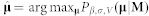 under the optimal values of the hyper-parameters *β*, *σ*, and *V*. Given the hyper-parameters *β*, *σ*, and *V*, this maximization can be readily performed using Newton's iteration method, as shown in Section S2 in Supplementary Information. Here, in this model, the detection rate during the period between the occurrence time *t*_0_ = 0 of the main shock and the time *t*_1_ of the first detected aftershock, *μ*(*t*) for *t*_0_ < *t* < *t*_1_ is set to *μ*(*t*) = *Μ*_0_, where *Μ*_0_ is the magnitude of the main shock.

The optimal estimate of the hyper-parameters *β*, *σ*, and *V* is obtained as follows. Here, we introduce a prior probability distribution *P*(*β*) for the *b*-value to avoid over-fitting the model and to achieve a good fit to the data for a short estimation period; it is given explicitly below. In this case, the hyper-parameters are optimized by maximizing the posterior probability distribution 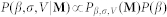, where 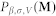 is the marginal likelihood function, given as 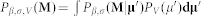. Because the exact treatment of the non-Gaussian high-dimensional integration appearing in the calculation of the marginal likelihood function is intractable, we approximate this optimization by the method described in Section S3 in Supplementary Information.

In this paper, the prior distribution *P*(*β*) is set to be Gaussian with the mean of 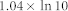 and standard deviation of 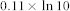. This agrees with the distribution of *β* estimated by using the Z-map^31^ for earthquakes with magnitude *M* ≥ 5.0 that occurred in the area before the main shock.

A similar Bayesian model for estimating the time-dependent detection rate was implemented using cubic spline^19^ or broken-line-type parameterization^20^. The present state-space representation of the Bayesian model enables adaptive updating of the prediction in real time. Another novelty of the proposed model is the introduction of the prior probability distribution of the *b*-value. Owing to this prior, the estimate is robust even for a very short learning period.

## Author Contributions

T.O. and Y.O. designed the research. T.O. conducted the analysis. T.O., Y.O., Y.H. and K.A. wrote the paper.

## Supplementary Material

Supplementary Information Supplementary Information

## Figures and Tables

**Table 1:** Summary of the method

[Estimation]
1. Estimating the time-varying detection rate and the G-R formula (Methods).
1.1 Choose initial values of the hyper-parameters *β*, *σ*, and *V.*
1.2 Evaluate the posterior function 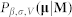 (Sec. S2).
1.3 Update the hyper-parameters (Sec. S3).
1.4 If the hyper-parameter converges, go to the step 2.
Else, back to the step 1.2.
2. Estimating the O-U formula by maximizing the likelihood function (Eq. 5).
[Forecast]
3. Calculate the expected rate of underlying aftershocks (Eq. 2), and the predictive interval (Sec. S1).

**Table 2:** Summary of estimated parameters. Parameters *b*, *K*, *p*, and *c* are defined in equation (1), *σ* is defined is in equation (3), and *V* is defined in equation (7)

Learning Period	*b*-value	σ	*V*	*K*	*p*	*c* (day)
0–3 h	1.17(± 0.10)	0.14(± 0.06)	1.11 × 10^−3^(± 6.97 × 10^−4^)	1.71 × 10^8^(± 9.19 × 10^7^)	1.29(± 0.19)	9.71 × 10^−3^(± 4.32 × 10^−4^)
0–6 h	1.25(± 0.09)	0.15(± 0.03)	4.01 × 10^−4^(± 2.34 × 10^−4^)	2.04 × 10^8^(± 5.64 × 10^7^)	1.60(± 0.12)	1.16 × 10^−2^(± 3.54 × 10^−4^)
0–12 h	1.24( ± 0.08)	0.28(± 0.02)	7.31 × 10^−6^(± 6.09 × 10^−6^)	3.45 × 10^8^(± 5.37 × 10^7^)	1.42(± 0.07)	6.19 × 10^−3^(± 1.99 × 10^−4^)
0–24 h	1.22( ± 0.07)	0.22(± 0.02)	6.64 × 10^−6^(± 4.60 × 10^−6^)	2.81 × 10^8^(± 2.92 × 10^7^)	1.29(± 0.05)	3.04 × 10^−3^(± 1.24 × 10^−4^)
