# Supplementary material for: Forecasting large aftershocks within one day after the main shock
Source: Sci Rep. 2013 Jul 17;3:2218. doi: 10.1038/srep02218 (PMC3715793; doi:10.1038/srep02218)
Supplement: Supplementary Information [file srep02218-s1.pdf]

Supplementary Information for ‘Forecasting large aftershocks within one day after the main shock’

Takahiro Omi[1,2*], Yosihiko Ogata[2,3], Yoshito Hirata[2], and Kazuyuki Aihara[2]

1. FIRST, Aihara Innovative Mathematical Modelling Project, Japan Science and Technology Agency, Kawaguchi, Saitama 332-0012, Japan
2. Institute of Industrial Science, The University of Tokyo, 4-6-1 Komaba, Meguro-ku, Tokyo 153-8505, Japan
3. The Institute of Statistical Mathematics, Tachikawa, Tokyo 190-8562, Japan

* Correspondence and requests for materials should be addressed to T.O. (email: omi@sat.t.u-tokyo.ac.jp)

S1. Prediction of the empirical occurrence rate

The number of future underlying aftershocks in a given time interval is predicted using the Reasenberg–Jones model in (1) of the main text. To make a reliable forecast from data with a small sample size, we take into account the uncertainty of the estimated parameters K, c, and p of the model, rather than using the maximum likelihood estimate of the parameters. For a given set of K, p, and c, the probability distribution $P(n|K,p,c)$ of the number n of underlying aftershocks with magnitude greater than M_p in time interval $[T_1, T_2]$ is given as the following Poisson distribution:

$$P(n|K,c,p) = \frac{f_{k,c,p}^{n} e^{-f_{k,c,p}}}{n!}, \tag{S1}$$

where $f_{k,c,p} = \int_{T_1}^{T_2} dt \int_{M_p}^{\infty} \frac{K}{(t+c)^p} 10^{-bM}$. The forecast of the probability distribution $P(n)$ of the number of underlying aftershocks is obtained by the weighted average of the probability distribution $P(n|K,c,p)$ over K, c, and p as follows:

$$P(n) = \frac{\int dK \int dp \int dc \; P(n|K,c,p)\, L(K,c,p)}{\int dK \int dp \int dc \;\; L(K,c,p)}, \tag{S2}$$

where the weight is proportional to the likelihood function $L(K,c,p)$.[33] To calculate the integrations in (S2) efficiently, we divide the p axis from 0.1 to 5.0 into a grid with intervals of 0.01. Then we replace integration over p with summation over all the grid points of p. We also replace integration over K and c by a single evaluation at K^* and c^*, respectively, which are obtained by maximizing

$$P(n) = \frac{\sum_p P(n|K^*,c^*,p) L(K^*,c^*,p)}{\sum_p L(K^*,c^*,p)}, \tag{S3}$$

where K^* and c^* are obtained for each grid point of p by maximizing the likelihood function with the fixed parameter p.

The 95% predictive interval $[f_1, f_2]$ of the empirical occurrence rate of the underlying aftershocks is plotted in Fig. 4 of the main text. This is obtained for the interval $[n_1/(T_2 - T_1), n_2/(T_2 - T_1)]$, where $[n_1, n_2]$ is the 95% confidential interval of $P(n)$.

S2. Maximization of the posterior function $P_{\beta,\sigma,V}(\boldsymbol{\mu}|\mathbf{M})$

The objective here is to estimate the mode $\hat{\boldsymbol{\mu}}$ of the posterior probability distribution

$\mathrm{P}_{\beta,\sigma,\mathrm{V}}(\boldsymbol{\mu}|\mathbf{M})$ (8). This is equivalent to maximizing $\ln P_{\beta,\sigma}(\boldsymbol{M}|\boldsymbol{\mu})P_V(\boldsymbol{\mu})$. From (6) and (7), we have

$$\ln P_{\beta,\sigma}(\boldsymbol{M}|\boldsymbol{\mu})P_V(\boldsymbol{\mu}) = \sum_{i=1}^{N} \ln\beta - \beta(M_i - \mu_i) - \frac{\beta^2\sigma^2}{2} + \ln\Phi(M_i|\mu_i,\sigma)$$
$$+ \sum_{i=1}^{N-2} -\frac{1}{2}\ln(2\pi V) - \frac{(\mu_{i+2} - 2\mu_{i+1} + \mu_i)^2}{2V} + const. \tag{S4}$$

Because $\ln P_{\beta,\sigma}(\boldsymbol{M}|\boldsymbol{\mu})P_V(\boldsymbol{\mu})$ is concave with respect to $\boldsymbol{\mu}$, this optimization can be readily performed using Newton's method: The posterior mode $\widehat{\boldsymbol{\mu}}$ is obtained by iteratively updating $\widehat{\boldsymbol{\mu}}$ according to the equation

$$\widehat{\boldsymbol{\mu}}^{(n+1)} = \widehat{\boldsymbol{\mu}}^{(n)} - \alpha H^{-1}\Delta\,, \tag{S5}$$

where Δ and H are the gradient and Hessian of $\ln P_{\beta,\sigma}(\boldsymbol{M}|\boldsymbol{\mu})P_V(\boldsymbol{\mu})$ at $\boldsymbol{\mu} = \widehat{\boldsymbol{\mu}}^{(n)}$, respectively, and α is the step size. The gradient and Hessian are given by

$$\nabla_{\boldsymbol{\mu}} \ln P_{\beta,\sigma}(\boldsymbol{M}|\boldsymbol{\mu})P_V(\boldsymbol{\mu}) = \mathrm{Vec}\left[\beta - \frac{\Psi(\widetilde{M}_i)}{\sigma}\right] - \frac{W\boldsymbol{\mu}}{V}, \tag{S6}$$

$$\nabla\nabla_{\boldsymbol{\mu}} \ln P_{\beta,\sigma}(\boldsymbol{M}|\boldsymbol{\mu})P_V(\boldsymbol{\mu}) = \mathrm{Diag}\left[-\frac{\Psi(\widetilde{M}_i)\left(\Psi(\widetilde{M}_i) + \widetilde{M}_i\right)}{\sigma^2}\right] - \frac{W}{V}\,, \tag{S7}$$

respectively, where we used the notation $\widetilde{M}_i = (M_i - \mu_i)/\sigma$, $\psi(x) = e^{-x^2/2}/\int_{-\infty}^{x} e^{-s^2/2}\,ds$, $\mathrm{Vec}[x_i] = (x_1, \dots, x_N)^T$, $\mathrm{Diag}[x_i] = \mathrm{diag}(x_1, \dots, x_N)$ and

$$W = \begin{pmatrix} 1 & -2 & 1 & 0 & \cdots & \cdots & 0 \\ -2 & 5 & -4 & \ddots & \ddots & & \vdots \\ 1 & -4 & 6 & \ddots & \ddots & \ddots & \vdots \\ 0 & \ddots & \ddots & \ddots & \ddots & \ddots & 0 \\ \vdots & \ddots & \ddots & \ddots & 6 & -4 & 1 \\ \vdots & & \ddots & \ddots & -4 & 5 & -2 \\ 0 & \cdots & \cdots & 0 & 1 & -2 & 1 \end{pmatrix}, \tag{S8}$$

with $W \in R^{N\times N}$. The update in (S5) is repeated until $\widehat{\boldsymbol{\mu}}^{(n)}$ converges.

S3. Expectation maximization method for optimizing the hyper-parameters

The hyper-parameters β, σ, and V are optimized using the EM method[32]. Here, we first consider the case that the prior $P(\beta)$ of the b-value is not employed. In this case,

the maximization of the marginal likelihood $P_{\beta,\sigma,V}(\boldsymbol{M}) = \int P_{\beta,\sigma}(\boldsymbol{M}|\boldsymbol{\mu})P_V(\boldsymbol{\mu})\, \boldsymbol{d\mu}$ yields the optimal estimates of the hyper-parameters. Then, the derived algorithm for the optimization is extended to the case that the prior $P(\beta)$ is employed.

The EM method provides a useful technique for optimizing a statistical model with hidden variables. It aims to find the hyper-parameters $\theta = \{\beta, \sigma, V\}$ that maximize the marginal likelihood function $P_\theta(\boldsymbol{M})$. The EM method iteratively updates the hyper-parameters as follows: (E-step) we evaluate the posterior distribution $P_{\theta_{old}}(\boldsymbol{\mu}\,|\boldsymbol{M})$ given the old hyper-parameter θ_{old}, and obtain the expectation $Q(\theta, \theta_{old})$ of the complete log likelihood function $\ln P_\theta\,(\boldsymbol{\mu}, \boldsymbol{M})$ over the posterior distribution $P_{\theta_{old}}(\boldsymbol{\mu}|\boldsymbol{M})$, given by

$$Q(\theta, \theta_{old}) = \int \boldsymbol{d\mu}\, P_{\theta_{old}}(\boldsymbol{\mu}|\boldsymbol{M}) \ln P_\theta(\boldsymbol{\mu}, \boldsymbol{M})\,. \tag{S9}$$

(M-step) The new hyper-parameter θ_{new} is determined by maximizing the expectation $Q(\theta, \theta_{old})$; that is, $\theta_{new} = \arg\max_\theta Q(\theta, \theta_{old})$. These E- and M-steps are repeated until the hyper-parameters converge.

From the relation $P_\theta(\boldsymbol{\mu}, \boldsymbol{M}) = P_{\beta,\sigma}(\boldsymbol{M}|\boldsymbol{\mu})P_V(\boldsymbol{\mu})$ and (S4), the expectation $Q(\theta, \theta_{old})$ is given as

$$\begin{aligned} Q(\theta, \theta_{old}) = \sum_i^N \ln\beta - \beta\big(M_i - E_{\theta_{old}}[\mu_i]\big) - \frac{\beta^2\sigma^2}{2} + E_{\theta_{old}}[\ln \Phi(M_i|\mu_i, \sigma)] \\ + \sum_{i=1}^{N-2} -\frac{1}{2}\ln(2\pi V) - \frac{E_{\theta_{old}}[(\mu_{i+2} - 2\mu_{i+1} + \mu_i)^2]}{2V} + const\,, \end{aligned} \tag{S10}$$

where $E_{\theta_{old}}[\cdot]$ represents the expectation over the posterior distribution $P_{\theta_{old}}(\boldsymbol{\mu}|\boldsymbol{M})$. Then, in the M-step, the new hyper-parameter V_{new} is obtained from the condition $(d/dV)Q(\theta, \theta_{old}) = 0$ as

$$\begin{aligned} V_{new} &= \frac{1}{N-2}\sum_{i=1}^{N-2} E_{\theta_{old}}[(\mu_{i+2} - 2\mu_{i+1} + \mu_i)^2] \\ &= \frac{1}{N-2} tr\big(E_{\theta_{old}}[\boldsymbol{\mu}\boldsymbol{\mu}^T]W\big). \end{aligned} \tag{S11}$$

Similarly, the new hyper-parameters β_{new} and σ_{new} are obtained by numerically solving the conditions $(d/d\beta)Q(\theta,\theta_{old}) = 0$ and $(d/d\sigma)Q(\theta,\theta_{old}) = 0$. These conditions lead to

$$\beta_{new} = \frac{-\widetilde{M} + \sqrt{\widetilde{M}^2 + 4\sigma_{new}^2}}{2\sigma_{new}^2}, \tag{S12}$$

$$\sum_{i=1}^{N}\left[-\beta_{new}^2\sigma_{new} - E_{\theta_{old}}\left[\psi\left(\frac{M_i - \mu_i}{\sigma_{new}}\right)\frac{M_i - \mu_i}{\sigma_{new}^2}\right]\right] = 0\,, \tag{S13}$$

where $\widetilde{M} = \sum_{i=1}^{N}\left(M_i - E_{\theta_{old}}[\mu_i]\right)/N$.

To calculate the expectations $E_{\theta_{old}}[\cdot]$ in (S11-13), we approximate the posterior function $P_{\theta_{old}}(\boldsymbol{\mu}|\boldsymbol{M})$ as the Gaussian with the mean equal to the posterior mode $\widehat{\boldsymbol{\mu}}$ and the covariance matrix equal to $C = -\left[\nabla\nabla_{\boldsymbol{\mu}} \ln P_{\beta,\sigma,V}(\boldsymbol{\mu}|\boldsymbol{M})\big|_{\boldsymbol{\mu}=\widehat{\boldsymbol{\mu}}}\right]^{-1}$. The mean and covariance matrix are obtained from (S5) and (S7). This Gaussian approximation is motivated by the log concavity of the posterior function $P_{\theta_{old}}(\boldsymbol{\mu}|\boldsymbol{M})$. Then we obtain

$$E_{\theta_{old}}[\mu_i] = \hat{\mu}_i, \tag{S14}$$

$$E_{\theta_{old}}[\boldsymbol{\mu}\boldsymbol{\mu}^T] = C + \widehat{\boldsymbol{\mu}}\widehat{\boldsymbol{\mu}}^T, \tag{S15}$$

$$E_{\theta_{old}}\left[\psi\left(\frac{M_i - \mu_i}{\sigma_{new}}\right)\frac{M_i - \mu_i}{\sigma_{new}^2}\right] = \int_{-\infty}^{\infty}\frac{d\mu_i}{\sqrt{2\pi C_{i,i}}}e^{-\frac{(\mu_i - \hat{\mu}_i)^2}{2C_{i,i}}}\psi\left(\frac{M_i - \mu_i}{\sigma_{new}}\right)\frac{M_i - \mu_i}{\sigma_{new}^2}. \tag{S16}$$

When we employ the prior distribution $P(\beta)$ for the b-values (β), the hyper-parameters are optimized by maximizing the posterior function of the hyper-parameters, $P(\beta,\sigma,V|\boldsymbol{M}) \propto P_{\beta,\sigma,V}(\boldsymbol{M})P(\beta)$. This can be also done using the EM method [p. 454 in ref. 25 in the main text]. In this case, the expectation $Q(\theta,\theta_{old})$ is modified as

$$Q(\theta,\theta_{old}) = \int \boldsymbol{d\mu}\, P_{\theta_{old}}(\boldsymbol{\mu}|\boldsymbol{M}) \ln P_\theta(\boldsymbol{\mu},\boldsymbol{M})\, P(\beta)\,. \tag{S17}$$

Here, we assume that the prior distribution $P(\beta)$ is the Gaussian with mean x_β and variance v_β. The introduction of the prior distribution $P(\beta)$ does not change the update rules (S11) and (S13) of the hyper-parameters, but the update rule (S12) is

modified as

$$\beta_{new} = \frac{-(N v_\beta \widetilde{M} - x_\beta) + \sqrt{\left(N v_\beta \widetilde{M} - x_\beta\right)^2 + 4 N v_\beta (N v_\beta \sigma_{new}^2 + 1)}}{2(N v_\beta \sigma_{new}^2 + 1)}. \quad \text{(S18)}$$

S4. Supplementary Discussion

A probability forecast of the seismic intensity in a disaster region is strongly desired. This forecast is possible if we use the Ishimoto–Iida formula[34] instead of the G-R formula. Namely, the frequency distribution of the maximum amplitude a of recorded seismic waves at a seismograph empirically follows power law distribution $\nu(a) = ka^{-m}$, and the value of the coefficient m is either estimated directly from maximum amplitude data (again deficiency of the data is expected) or use the relation to the b-value of the G-R formula as $m = b + 1$.[35] This formula, coupled with the O-U type function, is combined with the detection rate of aftershocks for forecasting in the early period. Once we determine a suitable station for a strong-motion seismograph or seismic intensity meter near the rupture source, we can directly predict the probability of a particular felt intensity by the proposed procedure.

References

32. Dempster, A. P., Laird, N. M. & Rubin, D. B. Maximum likelihood from incomplete data via the EM algorithm, *J. Roy. Stat. Soc. B* **39**, 1-38 (1977).

33. Akaike, H. On the use of the predictive likelihood of a Gaussian model, *Annals of the Institute of Statistical Mathematics* **32**, 311-324 (1980).

34. Ishimoto, M., & Iida, K. Observation of earthquakes registered with the microseissmograph constructed recently (I). *Bull. Earthq. Res. Inst. Tokyo Univ.* **17**, 443–478 (1939).

35. Asada, T., Suzuki, Z. & Tomoda, Y. Notes on the energy and frequency of earthquakes. *Bull. Earthq. Res. Inst. Tokyo Univ.* **29**, 289–293 (1951).

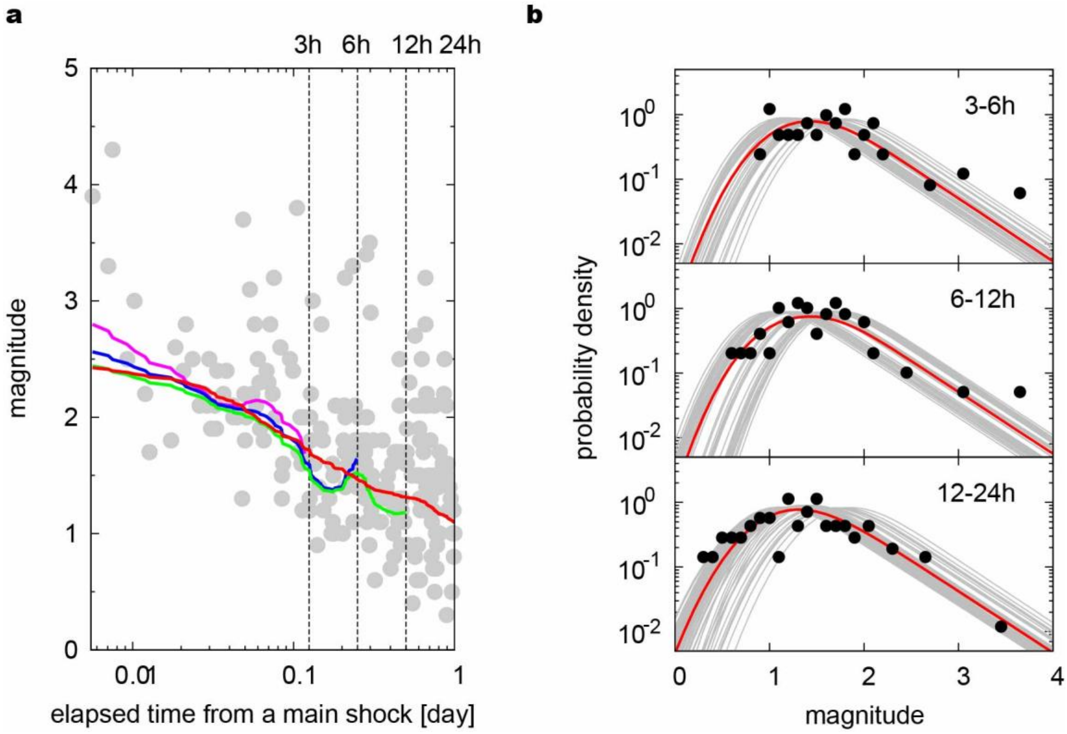

Figure S1 | Estimation of the time-dependent detection rate for the Hi-net catalog. Notations are the same as in Fig. 3 of the manuscript.

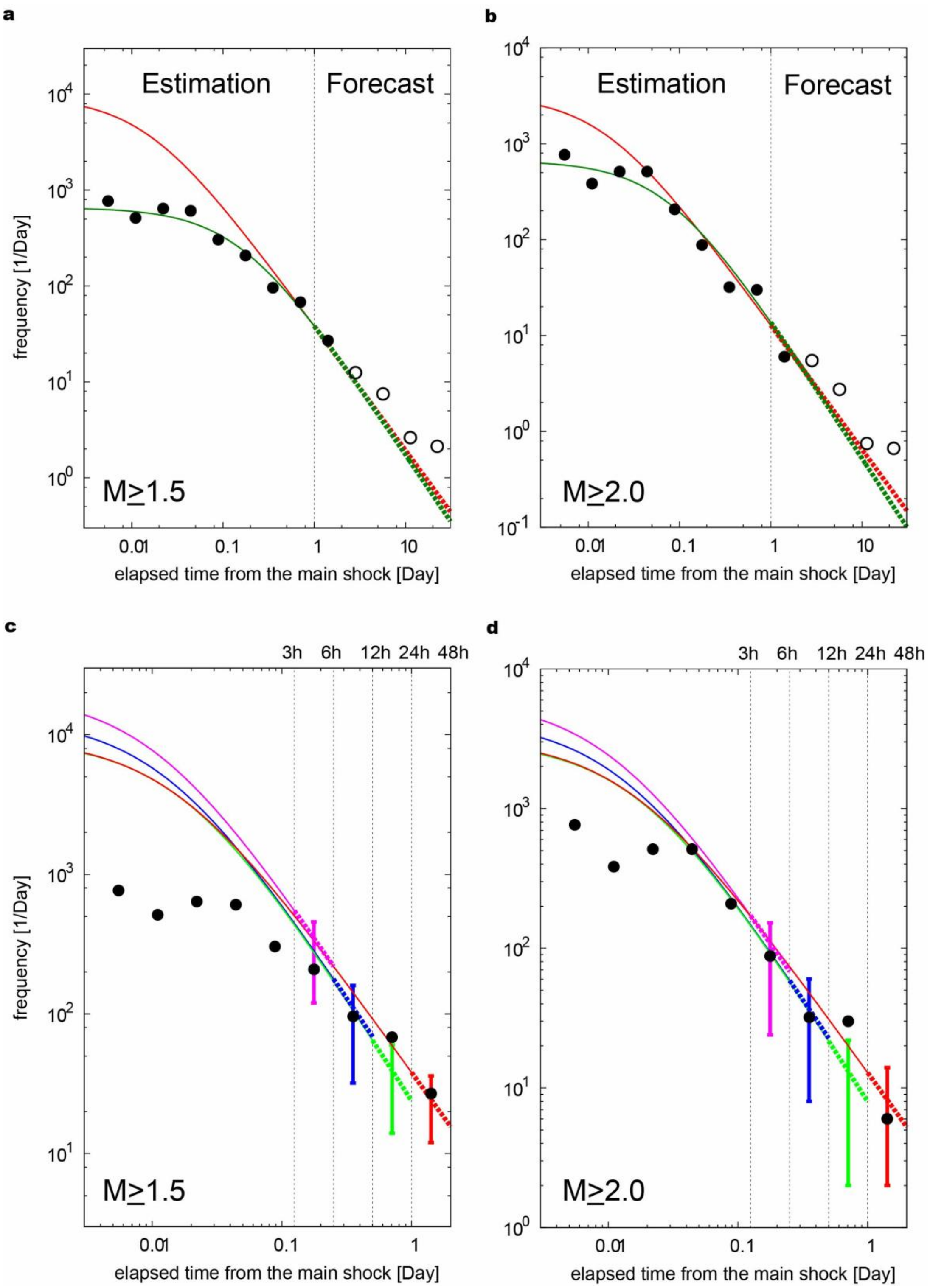

Figure S2 | Forecast of underlying aftershock activity for the Hi-net catalog. Notations are the same as in Fig. 4 of the manuscript except for that the closed (open) circles in (a) and (b) represents the empirical occurrence rate of the detected aftershocks

estimated based on the Hi-net (JMA) data.

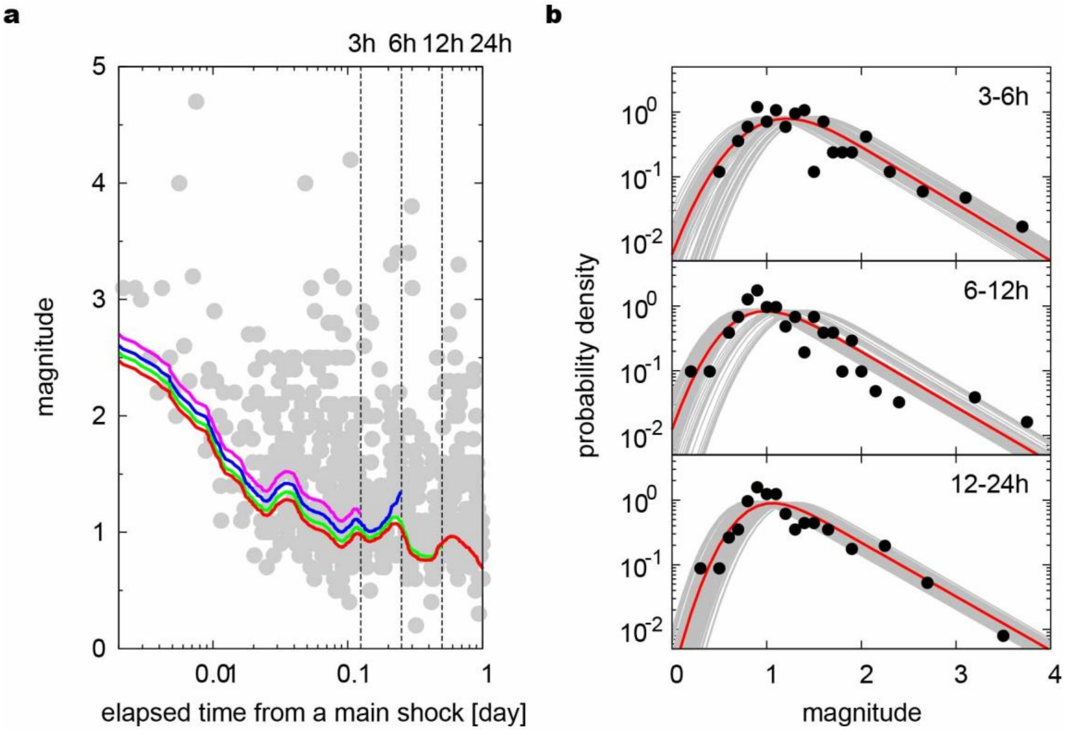

Figure S3 | Estimation of the time-dependent detection rate for the JMA catalog. Notations are the same as in Fig. 3 of the manuscript.

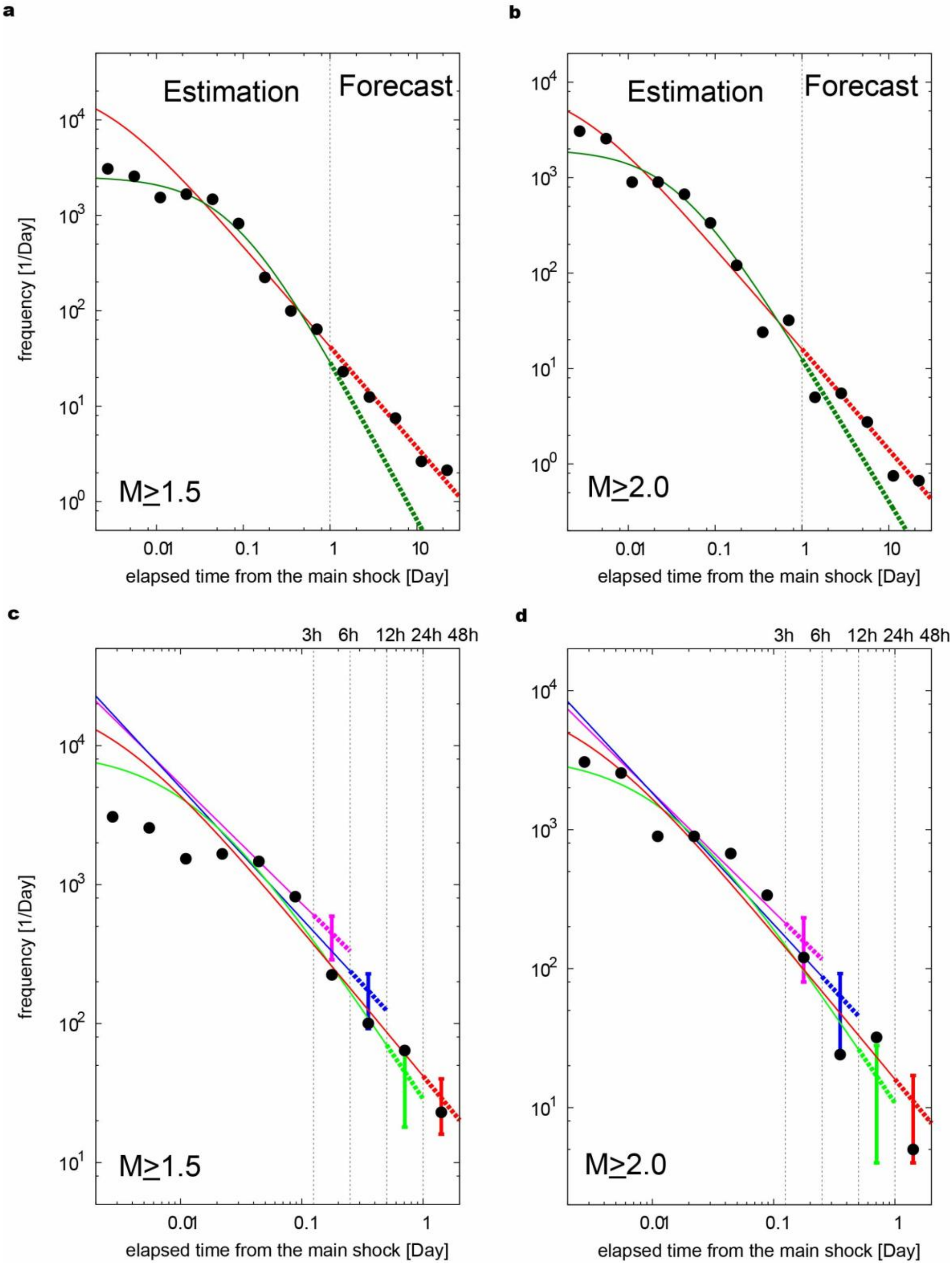

Figure S4 | Forecast of underlying aftershock activity for the JMA catalog. Notations are the same as in Fig. 4 of the manuscript.

Learning Period	*b*-value	σ	*V*	*K*	*p*	*c* (day)
0–3 h	1.01<br>(± 0.10)	0.34<br>(± 0.06)	1.19×10^{-4}<br>(± 2.45×10^{-4})	2.26×10^{3}<br>(± 1.49×10^{3})	1.40<br>(± 0.23)	1.06×10^{-2}<br>(± 3.48×10^{-4})
0–6 h	0.96<br>(± 0.09)	0.32<br>(± 0.05)	4.27×10^{-5}<br>(± 7.06×10^{-5})	1.49×10^{3}<br>(± 5.10×10^{2})	1.42<br>(± 0.14)	1.25×10^{-2}<br>(± 4.71×10^{-4})
0–12 h	0.95<br>(± 0.08)	0.29<br>(± 0.04)	2.49×10^{-5}<br>(± 4.10×10^{-5})	1.30×10^{3}<br>(± 2.74×10^{2})	1.48<br>(± 0.10)	1.81×10^{-2}<br>(± 6.79×10^{-4})
0–24 h	0.95<br>(± 0.08)	0.35<br>(± 0.04)	8.37×10^{-7}<br>(± 1.20×10^{-6})	2.00×10^{3}<br>(± 2.52×10^{2})	1.30<br>(± 0.07)	1.47×10^{-2}<br>(± 6.45×10^{-4})

Table S1 | Summary of estimated parameters for the Hi-net catalog.

Learning Period	*b*-value	σ	*V*	*K*	*p*	*c* (day)
0–3 h	0.91<br>(± 0.08)	0.34<br>(± 0.04)	8.78×10^{-6}<br>(± 6.37×10^{-6})	4.38×10^{3}<br>(± 1.12×10^{3})	0.86<br>(± 0.08)	8.88×10^{-7}<br>(± 1.52×10^{-7})
0–6 h	0.87<br>(± 0.07)	0.30<br>(± 0.04)	6.70×10^{-6}<br>(± 4.35×10^{-6})	2.37×10^{3}<br>(± 4.11×10^{2})	0.94<br>(± 0.06)	7.96×10^{-6}<br>(± 8.56×10^{-7})
0–12 h	0.85<br>(± 0.06)	0.26<br>(± 0.03)	6.01×10^{-6}<br>(± 3.58×10^{-6})	9.92×10^{2}<br>(± 1.47×10^{2})	1.31<br>(± 0.06)	1.23×10^{-2}<br>(± 6.77×10^{-4})
0–24 h	0.84<br>(± 0.05)	0.23<br>(± 0.02)	4.54×10^{-6}<br>(± 2.57×10^{-6})	1.32×10^{3}<br>(± 1.23×10^{2})	1.06<br>(± 0.04)	2.38×10^{-3}<br>(± 1.90×10^{-4})

Table S2 | Summary of estimated parameters for the JMA catalog.
